# Increasing Incidence of Human Melioidosis in Northeast Thailand

**DOI:** 10.4269/ajtmh.2010.10-0038

**Published:** 2010-06

**Authors:** Direk Limmathurotsakul, Surasakdi Wongratanacheewin, Nittaya Teerawattanasook, Gumphol Wongsuvan, Seksan Chaisuksant, Ploenchan Chetchotisakd, Wipada Chaowagul, Nicholas P.J. Day, Sharon J. Peacock

**Affiliations:** Department of Tropical Hygiene and Mahidol-Oxford Tropical Medicine Research Unit, Faculty of Tropical Medicine, Mahidol University, Bangkok, Thailand; Department of Microbiology, Faculty of Medicine, Khon Kaen University, Khon Kaen, Thailand; Department of Microbiology, Sappasithiprasong Hospital, Ubon Ratchathani, Thailand; Department of Medicine, Khon Kaen Hospital, Khon Kaen, Thailand; Faculty of Medicine, Khon Kaen University, Khon Kaen, Thailand; Department of Medicine, Sappasithiprasong Hospital, Ubon Ratchathani, Thailand; Center for Clinical Vaccinology and Tropical Medicine, Nuffield Department of Clinical Medicine, University of Oxford, Churchill Hospital, Oxford, United Kingdom; Department of Microbiology and Immunology, Faculty of Tropical Medicine, Mahidol University, Bangkok, Thailand; Department of Medicine, Cambridge University, Addenbrooke's Hospital, Cambridge, United Kingdom

## Abstract

Melioidosis is a serious community-acquired infectious disease caused by the Gram-negative environmental bacterium *Burkholderia pseudomallei*. A prospective cohort study identified 2,243 patients admitted to Sappasithiprasong Hospital in northeast Thailand with culture-confirmed melioidosis between 1997 and 2006. These data were used to calculate an average incidence rate for the province of 12.7 cases of melioidosis per 100,000 people per year. Incidence increased incrementally from 8.0 (95% confidence interval [CI] = 7.2–10.0) in 2000 to 21.3 (95% CI = 19.2–23.6) in 2006 (*P* < 0.001; χ^2^ test for trend). Male sex, age ≥ 45 years, and either known or undiagnosed diabetes were independent risk factors for melioidosis. The average mortality rate from melioidosis over the study period was 42.6%. The minimum estimated population mortality rate from melioidosis in 2006 was 8.63 per 100,000 people (95% CI = 7.33–10.11), the third most common cause of death from infectious diseases in northeast Thailand after human immunodeficiency virus (HIV)/acquired immunodeficiency syndrome (AIDS) and tuberculosis.

## Introduction

Melioidosis is a severe community-acquired infectious disease caused by the Gram-negative bacillus *Burkholderia pseudomallei*. This organism is present in the environment in a defined geographic distribution, and infection is acquired through bacterial inoculation or contamination of wounds, inhalation, and ingestion.^1^ Melioidosis is most frequently reported from northeast Thailand where it is the most common cause of community-acquired bacteremia,^2^ and from Darwin, northern Australia where it is the most common cause of fatal community-acquired septicemic pneumonia.^3^ Melioidosis also occurs across much of South and East Asia and parts of South America;^4^,^5^ infection may be grossly underreported in these areas, because diagnostic confirmation relies on a microbiological culture that is often unavailable in resource-restricted regions of the world. Infection in adulthood usually occurs in people with one or more pre-disposing factors, and the strongest risk factor is diabetes mellitus.^2^,^6^ Overall mortality has been reported to be 50% in northeast Thailand and 19% in Australia,^7^,^8^ a difference most likely caused by the inequity in availability of intensive-care facilities.^9^

A previous study performed in northeast Thailand reported the incidence of melioidosis to be relatively constant at 4.4 per 100,000 people per year (95% confidence interval [CI] = 3.8–5.0) between 1987 and 1991.^2^ This is low compared with the recently reported incidence rates of 19.6 and 20.0 per 100,000 people per year in northern Australia and Papua New Guinea, respectively.^6^,^10^ The study reported here was undertaken in response to growing suspicions among clinicians and researchers in northeast Thailand that the incidence of melioidosis is increasing over time. Our objectives were to recalculate the incidence rate of melioidosis in this region, evaluate factors associated with the varying incidence rate, and compare the rate of death from melioidosis with other infectious diseases in our population.

## Materials and Methods

### Study site.

Northeast Thailand has a population of 21.4 million (Department of Provincial Administration, Thailand, unpublished data), and it consists of 19 provinces and covers 170,226 km^2^. Ubon Ratchathani is the largest province in northeast Thailand with a population of 1.8 million, covers 16,112 km^2^, and is bordered by Cambodia to the south and Laos to the east. Sappasithiprasong Hospital, the largest hospital in the province and a referral center for 25 community hospitals, served as the study site. This is the major referral center in the province for cases of melioidosis.

### Identification of study population.

We undertook active surveillance to identify all patients presenting to Sappasithiprasong Hospital with melioidosis between January 1, 1997 and December 31, 2006 as part of clinical trials conducted at this study site. The hospital diagnostic laboratory was contacted daily to identify patients with one or more cultures positive for *B. pseudomallei*. Rounds of the medical and intensive-care wards were conducted daily to identify additional cases. Any patients not already known to us who were suspected of having melioidosis based on presenting clinical features had samples taken for culture, including blood, throat swab, sputum (or tracheal aspirate), pus and surface swab from wounds and skin lesions. All patients with microbiologically confirmed melioidosis were visited daily to obtain information on history, examination findings, and outcome, which was defined as survival to discharge or in-hospital death. A minority of relatives take moribund patients home from this hospital to die, and these cases were assumed to have died. Ethical permission for all clinical trials was obtained from the Ethical and Scientific Review Subcommittee of the Thai Ministry of Public Health.^11^–^14^

### Data analyses.

Patient data used in this study were age, gender, presence of diabetes mellitus, and death. Patients with diabetes were categorized into two groups: (1) the patient had a pre-existing history of diabetes, or (2) the patient did not have a history of diabetes but had hyperglycemia at the time of admission with melioidosis. The latter group contained patients that survived to discharge and were confirmed as new diabetics, and those that died before diabetes could be confirmed or refuted. Patients who had acute hyperglycemia at presentation that survived and were not diabetic on subsequent testing were classified as non-diabetics. Hemoglobin A1c values were not available. The Third National Health Examination Survey 2004 was used to obtain estimates on the prevalence of diagnosed and undiagnosed diabetes in the Thai population.^15^ Population data by age and sex for Ubon Rathchathani province for the years 1997–2006 were obtained from the Department of Provincial Administration, Thailand. The overall, age-stratified, and sex-stratified annual incidences of melioidosis in Ubon Ratchathani province were calculated as the number of culture-confirmed cases identified at Sappasithiprasong Hospital per 100,000 people. The annual population mortality rate from melioidosis in Ubon Ratchathani province was calculated as the number of patients with melioidosis who died at Sappasithiprasong Hospital per 100,000 people. Data on population mortality rates for other infectious diseases in Thailand were obtained from the Ministry of Public Health.^16^ Data on annual rainfall in Ubon Ratchathani in the years 1997–2006 were obtained from the Thai Meteorological Department. Univariable and multivariable Poisson regression models were used to calculate crude and adjusted rate ratios. CIs were calculated using the Poisson method. The Spearman correlation coefficient was used to evaluate the association between the incidence of melioidosis and annual rainfall. A χ^2^ test for trend was used to assess change in proportion over time. All analyses were performed using STATA version 10.1 (Stata Corp., College Station, TX).

## Results

### Incidence of melioidosis.

A total of 2,243 patients were admitted to Sappasithiprasong Hospital between 1997 and 2006 with their first episode of culture-confirmed melioidosis. Overall, 24% of cases were diagnosed as a result of active case finding on the medical and intensive-care wards. The average annual incidence rate for the province during the 10-year study period was 12.7 cases per 100,000 people per year, but the rate showed considerable variability over time (Table 1). A year-on-year increase was observed between 2000 and 2006, and an annual incidence (100,000 people) of 8.0 (95% CI = 7.2–10.0) in 2000 rose to 21.3 (95% CI = 19.2–23.6) in 2006 (*P* < 0.001; χ^2^ test for trend). There was a negative association between the total annual rainfall and the number of melioidosis cases in each year (Spearman's ρ = −0.89; *P* < 0.001) (Table 1).

### Incidence of melioidosis by age, gender, and diabetes.

Twenty-seven patients were excluded from this analysis, because the history of underlying diseases was not recorded. Of the 2,217 patients remaining, 1,296 (58.5%) were male, and 921 (41.5%) were female. Median age was 49 years (interquartile range [IQR] = 37–60 years), and 199 patients (9.0%) were younger than 15 years of age. A total of 662 patients (29.9%) were patients with known diabetes, and an additional 370 patients (16.7%) were defined as having previously undiagnosed diabetes at presentation with melioidosis. The previously undiagnosed diabetes group was made up of 222 patients (60%) who survived their episode of melioidosis and had confirmed diabetes and 148 patients (40%) who were hyperglycemic at presentation but died of melioidosis before the presence of diabetes could be investigated. Table 2 shows the average annual incidence rate of melioidosis by age, sex, and presence of diabetes. Melioidosis was more common in males, and incidence was highest in the 55- to 64-year age group. Crude and adjusted rate ratios (RR) were calculated for gender, age, and diabetes (Table 3). Male sex, age ≥ 45 years, and both known and undiagnosed diabetes were independent risk factors for melioidosis. Known and undiagnosed diabetes were associated with an adjusted RR of 12.4 and 7.8, respectively, compared with no diabetes (*P* < 0.001).

### Deaths from melioidosis.

**Figure 1.**
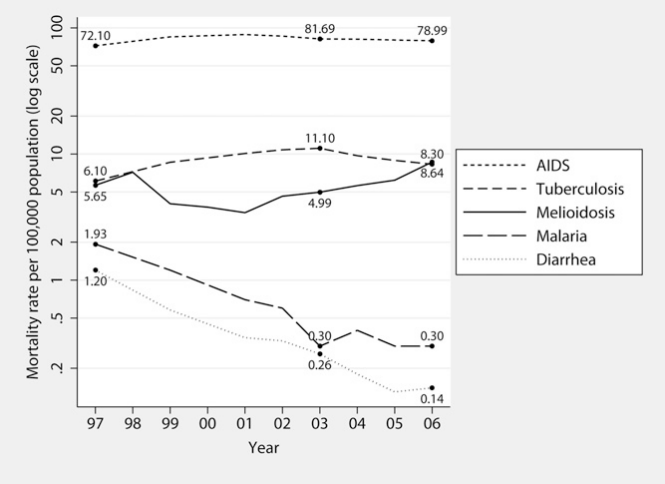
Mortality rates from infectious diseases per 100,000 people in Ubon Ratchathani province between 1997 and 2006. Mortality rates due to AIDS, tuberculosis, malaria and diarrhea were taken from the Thailand health profile report 2005–2007 from the Ministry of Public Health Thailand.^16^

Death occurred in 956 of 2,243 patients, and the average mortality rate over the study period was 42.6% (Table 1). There was a decrease in mortality rate over time from 49% in 1997 to 40.5% in 2006 (*P* = 0.006; χ^2^ test for trend). The average mortality rate from melioidosis was calculated for the province as 5.41 per 100,000 people per year. Figure 1 shows a comparison between these data and national population mortality rates attributed to other infectious diseases. If national data for deaths from infectious diseases are representative for Ubon Ratchathani province, melioidosis was the third most common cause of death from an infectious disease over the study period after human immunodeficiency virus (HIV)/acquired immunodeficiency syndrome (AIDS) and tuberculosis. In 2006, the population mortality rate attributed to melioidosis was comparable with the mortality rate attributed to tuberculosis (8.64 [95% CI = 7.33–10.11] versus 8.30 per 100,000 people per year, respectively).

## Discussion

Our data suggest that melioidosis is the third most common cause of death from an infectious disease in our region after HIV/AIDS and tuberculosis. Deaths from melioidosis and tuberculosis were comparable in 2006, and melioidosis may become established as the second most common cause of death if the current trend of increasing melioidosis incidence is sustained. The population mortality rate for melioidosis is more than from malaria and diarrheal illness combined in this setting, diseases that are usually considered to be of high priority by funding agencies and global health organizations. We propose that the number of cases and deaths from melioidosis reported here represents a minimum estimate, because we have not accounted for patients presenting to community hospitals in the province who were not referred to Sappasithiprasong Hospital. There are several reasons for the lack of referral, including patients with mild disease and those who were seriously ill and died shortly after admission to a community hospital or during inter-hospital transfer. Diagnostic microbiology is not available in these hospitals, and it is also possible that the diagnosis could be missed. Melioidosis can present with a wide range of clinical manifestations, and our study shows that active use of diagnostic microbiology can improve case detection. It is also likely that melioidosis is underreported as a cause of death in the National Statistics; laboratory isolation and identification of *B. pseudomallei* may take up to 1 week, and one-half of deaths because of melioidosis occur in the first 48 hours after presentation to hospital when the true diagnosis may be unknown and not listed on the death certificate.

The death rate from melioidosis showed a modest decline during the study period and is currently around 40%. This improvement was not associated with changes in recommended antimicrobial regimens over the study period. Ceftazidime has been the recommended first-line parenteral therapy at Sappasithiprasong Hospital for suspected or proven melioidosis after a study published in 1989 showed ceftazidime to be associated with a 50% overall reduction in mortality compared with a combination of chloramphenicol, doxycycline, and trimethoprim-sulphamethoxazole.^17^ Improved outcome from melioidosis over the study period may reflect improvements in the standard of medical care provided in provincial Thailand. However, the death rate of 40% is double that reported for patients with melioidosis at the Royal Darwin Hospital in Northern Australia.^7^ The major cause of death in our melioidosis patients is severe sepsis and the associated organ failure. Most cases are not treated in an intensive-care setting because of limited resources, and we propose that improvements in outcome would require investment in early sepsis management and critical-care facilities. Evidence for the efficacy of improved intensive-care unit management for patients with melioidosis comes from the experience described at the Royal Darwin Hospital.^9^ There is also anecdotal evidence from Khon Kaen University Hospital in neighboring Khon Kaen province where patients with severe melioidosis are treated in an intensive-care unit wherever possible and the mortality rate is around 20% (P. Chetchotisakd, personal communication).

There are several possible explanations for the increasing rate of melioidosis over time. Our method of case ascertainment did not change during the study, but most patients admitted to Sappasithiprasong Hospital were referred from community hospitals situated throughout the province; it is possible that referral patterns have changed over this period. A rise in incidence could also relate to rising life expectancy and a concomitant increase in people with pre-disposing conditions. Data available for life expectancy in Thailand between 1998 and 2004 show a modest increase from 68.9 years in 1998 to 70.3 years in 2004.^16^ The strongest risk factor for melioidosis is diabetes mellitus, which was shown in this study to put individuals at vastly increased risk of *B. pseudomallei* infection. The prevalence of diabetes in people in Thailand seems to be rising over time, and the reported rates of diagnosed plus undiagnosed diabetes are 2.3% for 1991, 4.6% for 1996, and 6.7% for 2004.^15^,^16^ The adjusted RR of melioidosis for diabetes of 12.4 in this study is consistent with that reported from northern Australia.^6^ We are not aware of any factors relating to changes in social behavior that would lead to an increase in the rate or duration of exposure to *B. pseudomallei* in the environment, and no association was found with annual rainfall. Understanding the reasons behind the increasing incidence in melioidosis is important for targeted prevention, and it is the basis of on-going studies.

A limitation of this study is that the incidence of melioidosis and population mortality rates were based on active surveillance at a single hospital. This provides a minimum estimate for a single province and may not be accurate for the rest of northeast Thailand. To address this, we contacted 19 provincial hospital laboratories in northeast Thailand to obtain data on the number of culture-confirmed cases of melioidosis in 2007. A total of 1,865 culture cases were defined without active surveillance, including 387 patients defined from Ubon Ratchathani provincial hospital laboratory. This is equivalent to an annual incidence rate for northeast Thailand of 8.7 per 100,000 people (95% CI = 8.3–9.1 per 100,000 people), which is a minimum estimate that is significantly higher than the rate reported previously.^2^ Melioidosis is known to occur in the areas of Laos and Cambodia that are immediately adjacent to northeast Thailand,^18^–^20^ but incidence rates are poorly defined and may be grossly underestimated because of a lack of diagnostic microbiology facilities. We predict that melioidosis will become recognized as a major pathogen throughout this region in the wake of laboratory strengthening and use of standard guidelines for the investigation of suspected melioidosis.

## Figures and Tables

**Table 1:** Incidence of melioidosis and associated death rate between 1997 and 2006

Year	Average annual rainfall (mm) in Ubon Ratchathani province	Melioidosis patients (*N*)	Deaths	Mortality rate	Incidence rate per 100,000 people	Mortality rate per 100,000 people
1997	1,555.1	198	97	49.0%	11.53	5.65
1998	1,318.3	257	124	48.2%	14.85	7.16
1999	1,582.5	173	71	41.0%	9.83	4.04
2000	1,844.6	141	67	47.5%	7.98	3.79
2001	1,709.4	152	61	40.1%	8.54	3.43
2002	1,677.3	184	83	45.1%	10.26	4.63
2003	1,560.4	235	90	38.3%	13.02	4.99
2004	1,471.1	250	99	39.6%	14.18	5.62
2005	1,323.0	273	110	40.3%	15.38	6.20
2006	1,526.7	380	154	40.5%	21.31	8.64

Spearman correlation coefficient between the number of patients and the level of annual rainfall = −0.89; *P* < 0.001.

**Table 2:** Average incidence rate of melioidosis by age, sex, and diabetes

Population	Cases in this study	Total population*	Annual incidence of melioidosis (per 100,000 people)
Total population	2,217	1,745,364	12.7
Sex			
Female	921	875,727	10.5
Male	1,296	869,638	14.9
Age (years)			
< 15	199	418,459	4.8
15–24	71	315,076	2.3
25–34	193	341,134	5.7
35–44	402	272,645	14.7
45–54	528	181,646	29.1
55–64	503	110,835	45.4
65–74	248	68,691	36.1
> 75	73	36,878	19.8
Diabetes			
No diabetes	1,123	1,656,090	6.8
Known diabetes	662	45,448	145.7
Undiagnosed diabetes	370	43,826	84.4

* Average population in Ubon Ratchathani province for the period from 1997 to 2006.

**Table 3:** Crude and adjusted RR for melioidosis by age, sex, and diabetes

Risk factor	Crude RR	95% CI	*P* value	Adjusted RR	95% CI	*P* value
Sex						
Female	1.0		< 0.001	1.0		< 0.001
Male	1.4	1.3–1.5		1.5	1.4–1.6	
Age (years)						
< 45	1.0		< 0.001	1.0		< 0.001
≥ 45	4.7	4.3–5.2		2.8	2.6–3.1	
Diabetes						
No diabetes	1.0		< 0.001	1.0		< 0.001
Known diabetes	18.1	16.4–20.0		12.4	11.2–13.7	
Undiagnosed diabetes	10.3	9.1–11.6		7.8	6.9–8.9	
